# A Note on the Magnitude of the Feedback Effect in Environmentally Extended Multi-Region Input-Output Tables

**DOI:** 10.1111/jiec.12658

**Published:** 2017-09-06

**Authors:** Daniel Moran, Richard Wood, João F. D. Rodrigues

**Affiliations:** 1 Program for Industrial Ecology, Department of Energy and Process Engineering, NTNU, Sem Sælands vei 7, NO-7491 Trondheim, Norway; 2 Institute of Environmental Sciences CML, Leiden University, Leiden, the Netherlands

**Keywords:** environmental input-output analysis, feedback effects, hybrid life cycle assessment, imports, industrial ecology, input-output analysis (IOA)

## Abstract

Global multiregion input-output (MRIO) tables have been developed to capture international spillover effects due to demand in one country and production in other countries. International spillovers have been growing and have become so dominant, especially in environmental analysis, that their inclusion is essential when analyzing impacts of consumption. MRIO tables give full coverage of the world economy, but do not always respect the official data of a given country. When international spillovers also cause increased production in the country of demand, we see what are known as “feedback effects.” As coupled models are being developed that make use of an official foreground national input-output table (IOT) alongside an existing global MRIO, we are left in the situation where a coupled model does not use the official foreground information when modeling international feedback loops. The question thus arises: How large are these feedback loops for different environmental impacts? We look specifically at the amount of domestic production that is embodied in imports back into that region. We find that for emissions, the feedbacks are small, usually <2% of the total import footprint, though up to 6%+ for some countries in some years for some stressors. Our findings suggest that using Leontief multipliers from available MRIOs may be an acceptable method for modeling imports into national IOTs for environmentally extended MRIO analysis.

## Introduction

Environmental footprints or consumption-based accounts are increasingly being used in both academic work and policy to understand developmental and trade issues (Liu et al, 2015) related to, among others, embodied flows of greenhouse gases (Wiedmann 2009; Peters et al. 2012), appropriation of land use (Weinzettel et al. 2013), biodiversity (Lenzen et al. 2012b), and resource decoupling (Wiedmann et al. 2015). Global multiregional input-output (MRIO) tables (Miller and Blair 2009) are the data sets most commonly used for quantifying these footprints of consumption (Tukker et al. 2016), as they describe the economic transactions between different sectors in different countries and associated environmental impacts (sometimes called stressors), giving explicit coverage of international supply chains.

One advantage of MRIO tables is that they are able to capture both the direct and indirect international spillover effects. An example of a direct international spillover is the economic production of steel production in China due to vehicle production in Germany. An example of an indirect international spillover would be the mining of coal in Australia, which serves as an input into the steel production in China. As global production chains become more and more complex, these indirect supply chains are becoming more and more prevalent (see Wood et al. [2018], this issue). Modeling these supply chains using the unique production technologies of each region involved is a strong benefit of using MRIOs. One caveat of MRIO data is that data from different countries need to be harmonized and reconciled (Wood et al. 2014). As such, deviations occur between the statistical data of a single country and its representation in an MRIO database. Several recent papers have investigated the extent and reasons for divergence between national IO tables and various MRIOs tables currently available (Wilting 2012; Hoekstra et al. 2014; Inomata and Owen 2014; Moran and Wood 2014; Streicher and Stehrer 2014). The international spillover effects as represented by a MRIO model may not necessarily correspond to the expected level based on nationally reported imports and exports. Such a problem is not confined to MRIO data, but is also clearly apparent in the imbalance of global trade statistics (The Economist 2011). These deviations from statistical data of a single country can make it difficult for national policy making, as pointed out by Edens and colleagues (2015).

For national level policy and analysis, it can therefore be desirable to combine a foreground national (or “canonical”) input-output table (IOT) of superior data quality with other data that captures international spillovers, such as a global MRIO account for the rest of the world. This may be done for various reasons: to preserve sectoral detail in the national table, keep an official national table's values unaltered, or work with a national table that is omitted or not made explicit in an MRIO. There are various methods to perform this task that require inserting the new regional table and rebalancing the system, and additional processing steps.

We now mention briefly some recent notable efforts to build coupled models. Edens and colleagues (2015) have proposed the “Single Country National Accounts Consistent” (SNAC) method to prepare a multicountry MRIO that perfectly respects a given national IOT (i.e., values in the MRIO are identical to the corresponding value in the national table). In their method, the new foreground table overwrites the MRIO's equivalent table, and off-diagonal trade blocks are then rebalanced subject to the constraint that the newly introduced data remain untouched. The AISHA MRIO-building engine (Geschke 2012; Geschke et al. 2014) used to assemble the Eora MRIO (Lenzen et al. 2012a, 2013) is able to achieve essentially the same result, by introducing canonical national data for one country and setting the uncertainty of those values to zero so that those data are perfectly respected during the conflict resolution and balancing stage. Bachmann and colleagues (2014) and Wenz and colleagues (2014) have proposed methods to solve the related problem of incorporating a subnational MRIO in a global MRIO. Methodologically, this literature also covers the problem of adding a new country to a global MRIO since this country was originally part of a “Rest of the World” aggregate. More recently, Christis and colleagues (2016) study the magnitude of concordance, aggregation, and time errors in coupled models and provide guidance on trading off investments in improved modeling versus the reliability of results.

One simple approach for including international spillovers into national statistical models for consumption-based accounting is to use the multipliers from a global MRIO model and apply them to the vector of imports into the target country. Such approaches are common in life cycle assessment (Benini et al. 2014), material flow accounting (Schoer et al. 2012, 2013), and when looking at environmental impacts embodied in bilateral trade (EEBT) approaches of Peters and colleagues (Peters 2008; Peters et al. 2011), albeit the latter only captures direct international spillovers and excludes *all* indirect international spillovers. The advantage of these methods is in the ease of implementation. If the total environmental impact intensity of imported products can be captured through a background MRIO model, then this can be simply linked to the known level of imports in a national table. The disadvantage, however, is that in the EEBT approaches, indirect international spillovers are excluded to avoid double counting, while in the life cycle approaches, the indirect international spillovers are fully modeled in the global MRIO. This latter point can become relevant when what are known as “feedback effects” become important. International feedback effects are, for example, the economic output in Australia in coal mining, due to steel production in China due to demand for vehicles in Australia. This feedback effect may matter for tracing embodied environmental impacts. The magnitude of the feedbacks will vary across product types, and hence the net benefit of applying background MRIO multipliers depends on the empirical magnitude of those effects.

There is a large literature investigating feedback effects in the context of subnational MRIOs, starting with the seminal work of Miller (1966, 1969), who found out that feedback effects are negligible, thus suggesting that there is little value added in building expensive multiregional models. Naturally, regional and national economies are much more integrated presently, and this topic has been revisited many times, both from a theoretical (Round 1978, 1985; Gillen and Guccioni 1980; Miller 1986, Guccioni et al. 1988) and empirical angle (Europe by Sonis et al. [1993] and Dietzenbacher and van der Linden [1997], the United States by Sonis et al. [1995], Indonesia by Sonis et al. [1997], and Asia by Su and Ang 2011). Round (2001) provides a survey of this literature and suggests that the original conclusion of Miller (1966) remains unaffected.

To the best of our knowledge, a systematic analysis of the magnitude of feedback effects in the context of a global MRIO has never been performed, especially with the recent focus on environmental impacts embodied in trade. The impact of feedback effects has only been conceptually discussed by Kanemoto and colleagues (2012). Su and Ang (2011) published a study comparing EEBT and MRIO approaches in order to study feedback effects. What these researchers (and in the original publications on EEBT, e.g., Peters et al. [2011]) show is the magnitude of the indirect international spillover, including, but not limited to, the domestic feedback effect. That is, they capture emissions (in this case) that occur in all third-party countries from country of demand, while as far as we are aware, the common usage of feedbacks is the emissions that occur in the country of demand that is embodied in exports that are later reimported in products that have undergone external processing (see, e.g., Miller and Blair 2009, 80). While such studies are interesting to understand the impact of cutting off international supply chains in the life cycle impacts of consumption (see Peters 2008), they do not give insight into the potential differences in data, and hence footprint results inherent in linking national-level data to global MRIO databases. That is, there is no evidence to suggest whether the use of background MRIO multipliers are accurate or not in applying to national-level import data.

In this paper, we use the EXIOBASE3 global MRIO account (Stadler et al. 2018; for a recent overview of available databases, see Tukker and Dietzenbacher 2013) to investigate empirically how large these second-order flows are. We also explore how much these effects are changing over time.

## Methods and Data

Environmental footprints are calculated as the impact on the domestic territory plus impacts embodied in net imports, that is (equation 1):

1 $$ \boldsymbol{D}\kern0.33em =\boldsymbol{F}+\boldsymbol{M}-\boldsymbol{E} $$

where *F* is the territorial account, *M* represents impacts embodied in imports, and *E* represents impacts embodied in exports. Feedback emissions can be calculated relative to overall footprint *D*, or, relative to the total imports *M*. As discussed above, while there are differing definitions of feedback effects, what we are precisely interested in are the domestic impacts that are exported and reimported as embodied flows back to the domestic economy. This will give an indication on whether the (in)accuracy of the domestic MRIO data to national statistical data will have an impact on the impacts embodied in imports.

To calculate the share of domestic emissions in final demand of a region, we do as follows. Consider a multiregional setting, with *n* regions and *m* sectors; let **s** be the row vector of emission intensities (e.g., carbon emissions per unit of total output per sector), and **s**^***d***^ the domestic only emissions; let **x** be the economic output; let **L** be the Leontief inverse matrix (i.e., entry L_*ij*_ specifies the total output in sector *i* stimulated by one unit of demand in *j*), and let ***y***^***m***^ be a vector of total imports of target country *r*. The total (global) output due to imports in target country *r* is **x = L*y***^***m***^, and the total (global) environmental (e.g., carbon) footprint of country *r's* imports is **sx**, while the total emissions from domestic sources are ${\mathbf{s}}^{\boldsymbol{d}}\mathbf{x}$. We take the ratio between the two to calculate the importance of domestic emissions in the total footprint of imports, that is, feedback emissions equal $\frac{{\mathbf{s}}^{\boldsymbol{d}}\mathbf{x}}{\mathbf{s}\mathbf{x}}$. Note, using a fully populated **L** matrix double counts intermediate imports, and methods such as the EEBT approach have been suggested to avoid such issues. As EEBT approaches isolate all indirect international spillovers and not just feedback effects, their application is not useful here. We refer to the earlier literature, such as Schoer and colleagues (2012), that discuss the application of multipliers to imports and exports, and note here that any double counting of intermediate imports in the production chain are in both numerator and denominator.

All calculations in this study use the 200-product EXIOBASE3 MRIO model (Stadler et al. 2018). The EXIOBASE3 MRIO provides a full intercountry IOT (Tukker et al. 2013; Wood et al. 2015) in a time series from 1995 to 2011 (and now-casted results to 2015, though these were not used in order to avoid using lower confidence source data). All results are for year 2011, except for the time-series analysis in figure 3 which covers 1995–2011 (in current-year prices; no deflation was applied). The EXIOBASE3 database was chosen because of its high sector disaggregation (200 sectors per country) and coverage of all major economies (41 countries covering >95% of global GDP, plus five other regions). The Eora MRIO (Lenzen et al. 2012a, 2013) covers more countries, but mostly at lower resolutions, and using heterogenous classifications, which makes sector-wise results comparisons between countries difficult.

## Results

We find that for most countries, with a few exceptions, the feedback loops contribute under 2% of the total consumption-based carbon dioxide (CO_2_) emissions account (table 1). Russia and the United States have slightly larger feedbacks (2.7% and 4.1%, respectively), and China stands out with 6% to 8% of its total consumption-based emissions comprised of emissions embodied in reimported via feedback loops. Returning to the opening research question, the dominant finding of the results is that the feedback effects are consistently small.

**Table 1:** Feedback emissions (how much of the embodied CO_2_ emissions in imports were originally emitted in the country)

Country	Feedback emissions	Country	Feedback emissions
Australia	0.5%	Malta	0.0%
Austria	0.2%	Mexico	0.8%
Belgium	0.3%	Netherlands	0.3%
Brazil	0.8%	Norway	0.4%
Bulgaria	0.4%	Poland	0.4%
Canada	1.1%	Portugal	0.1%
China	6.1%	Romania	0.3%
Croatia	0.1%	RoW Africa	1.1%
Cyprus	<0.1%	RoW America	1.1%
Czech Republic	0.6%	RoW Asia and Pacific	2.5%
Denmark	0.4%	RoW Europe	0.9%
Estonia	0.6%	RoW Middle East	3.5%
Finland	0.1%	Russia	3.0%
France	0.5%	Slovakia	0.2%
Germany	1.7%	Slovenia	<0.1%
Greece	0.2%	South Africa	0.8%
Hungary	0.1%	South Korea	0.6%
India	0.9%	Spain	0.4%
Indonesia	0.7%	Sweden	0.2%
Ireland	0.1%	Switzerland	0.1%
Italy	0.3%	Taiwan	0.4%
Japan	0.8%	Turkey	0.2%
Latvia	0.1%	United Kingdom	0.5%
Lithuania	0.1%	United States	4.2%
Luxembourg	<0.1%		

*Note*: Results based on the EXIOBASE3 MRIO. Results are for 2011.

CO_2_ = carbon dioxide; RoW = rest of the world.

It may be possible to divine some relationships between trade exposure, industry specialization, size of gross domestic product (GDP), and magnitude of feedback effects. Larger and/or more developed countries may expect to have fewer feedback loops since exports will be fully “digested” (processed into intermediate products) when they leave and will not return; or, those same countries could equally well expect more feedback loops as they participate in more, and more complex, supply chains. Similarly, we cannot predict a priori whether more trade-exposed countries can expect more, or less, feedback loops. In any case, while it is interesting to speculate on which countries have more feedback loops and why, we shall not pursue this question further here simply because we find that feedback effects are generally small, and for the few instances where they are big, understanding the underlying economics would not help practitioners build better coupled models.

**Figure 1:**
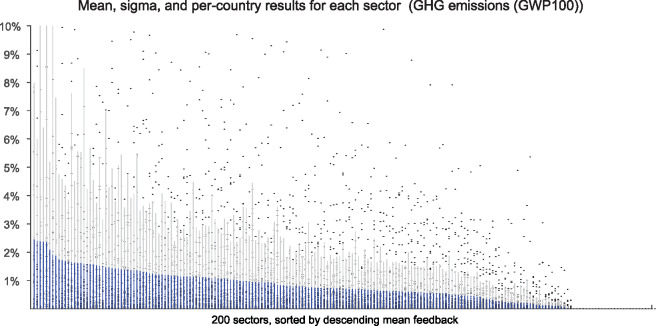
Feedback GHG emissions as share of total footprint of imports by sector of final consumption. The vertical axis shows the percentage of feedback emissions in final consumption, and the final consumption good, of which 200 are distinguished in EXIOBASE3, are arrayed in descending order of average feedback effect along the horizontal axis. Note that 17 products are purely intermediate and have zero final demand. Individual points show the sector-wise results across all 41 countries, solid blue lines show the mean in each sector, and the light gray lines show the standard deviation within each sector. GHG = greenhouse gas; GWP100 = global warming potential for 100-year time horizon.

We may explore the results at the sector level as well. Figure 1 shows the feedback CO_2_ emissions as share of total footprint, by sector of final consumption, averaged across all countries. All sectors have a mean fraction of feedback emissions of less than 2.5% with a standard deviation below 10%. Some products are purely intermediate goods and final consumption is zero; these products occupy the space at the far right end of the horizontal axis in figure 1. As seen in that figure, feedback loops appear to be well mixed across sectors and there are no individual sectors that stand out.

**Figure 2:**
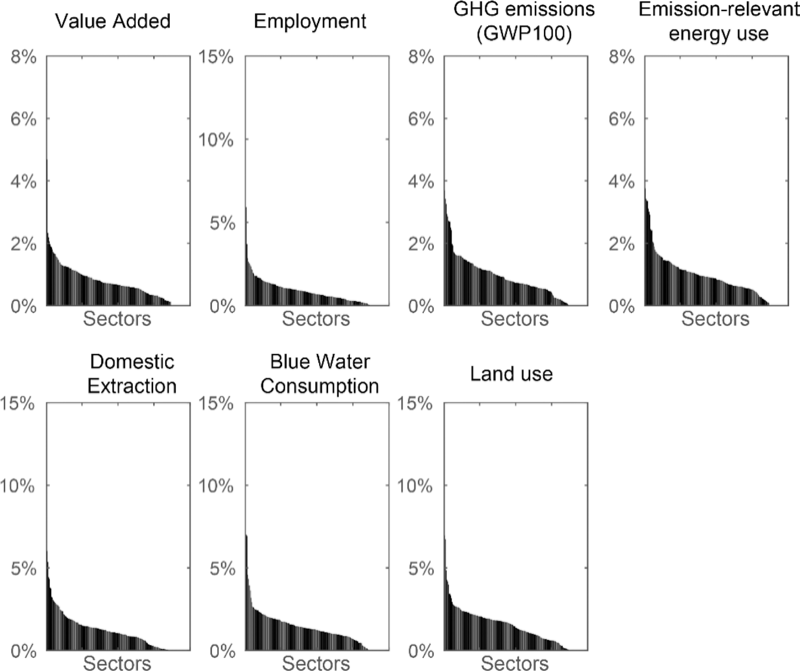
Same as figure 1 but across several different environmental stressors: average across countries of fraction of feedback emissions embodied in imports of different sectors for methane, water consumption, and cropland, ranked by decreasing order. Feedback effects will vary according to the mix of products considered, hence will be different for different stressors, but are small (<5% of imports) for most sectors and stressors considered. GHG = greenhouse gas; GWP100 = global warming potential for 100-year time horizon.

We investigated the results for other embodied environmental impact flows other than CO_2_ and found essentially similar results (figure 2). The magnitude of the feedback loops as a constituent of the footprint of imports is greater for other stressors than for CO_2_, but is still small. Using the environmental extensions from the EXIOBASE3 database, we investigated methane, virtual water, and embodied cropland. Feedback effects are slightly more pronounced for other embodied resource flows, but are still at low levels, comprising still generally <5% in each sector on average.

**Figure 3:**
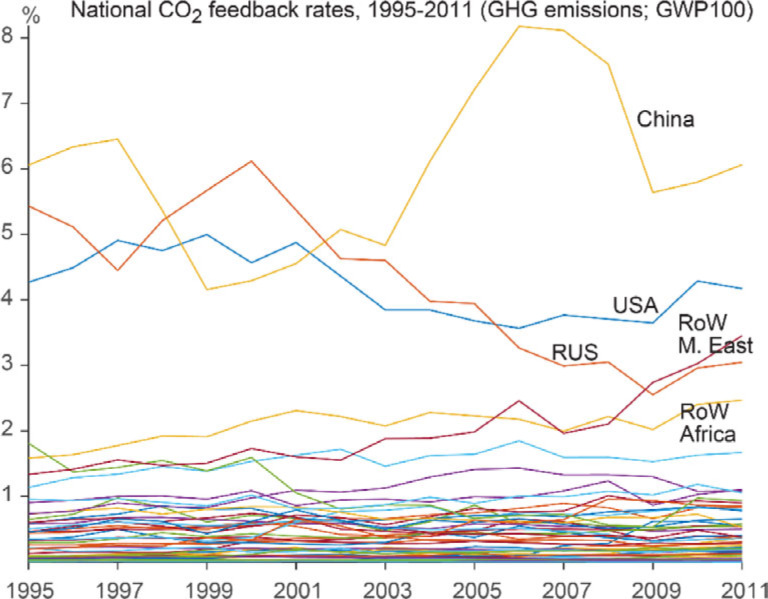
Fraction of each country's CO_2_ footprint of imports arriving via feedback loops, change over time. Horizontal axis shows total feedback emissions, horizontal axis shows time, and lines denote individual countries. For most countries, the share is small and stable over time. China and the United States stand out and are discussed further in the text. Countries <1% are not shown. CO_2_ = carbon dioxide; GHG = greenhouse gas; GWP100 = global warming potential for 100-year time horizon; RoW = rest of the world.

It is also worthwhile to investigate whether the magnitude of the feedback loops evolves over time, as the economic and trade structure changes. Figure 3 shows the results of such an analysis, presenting the share of total CO_2_ footprint arriving via feedback loops for different countries in the period 1995–2011. This share is small and stable over time for most countries, though a spike for China (up from ∼5% to 6% to 8% around 2007), the slow decline of feedbacks in the United States and Russia, and the slow growth of feedback effects in the Rest of World–Middle East and Rest of World–Africa regions do stand out as discernable features. The higher share of feedbacks for China is not surprising, as China is a major manufacturer and one of the largest, if not the largest, economy in the world, and the structure of its value chain evolved drastically during this period. There is an increasing amount of emissions from primary production in China that are embodied in exports and then processed into secondary and tertiary goods and services abroad, which are later re-imported and purchased by Chinese final consumers.

## Discussion and Conclusions

In this paper, we examined the magnitude of environmental feedback effects in a global MRIO. We found that, for most countries, feedback effects represent less than 2% of total embodied emissions, which is much smaller than the variation/uncertainty in current MRIO results (Moran and Wood 2014; Edens et al. 2015).

A potential limitation of this study is that a single MRIO was explored, and it is therefore worth asking whether the results would have differed significantly if an alternative MRIO had been used. Based on the inter-MRIO comparison reported in a special issue of this Journal in 2014 (Moran and Wood 2014) we expect that our basic finding—that feedback loops do not constitute major flows—would not. Another point which we did not pursue was to ask whether the results would hold in the context of endogenous or closed models. That is, we only examined the magnitude of interindustry indirect effects, but not the magnitude of induced effects, those that result from the additional expenditure which accrues from households’ income (Miyazawa 1968), capital turnover (Lenzen and Treloar 2004), or secondary flows in the social accounting matrix (Pyatt and Round 1985). It may happen that some of the largest feedbacks for some countries might arise from the incorporation of consumption-income loops, if interindustry linkages are weak relative to income-consumption linkages. We believe this to be a topic of great importance in our modern globalized world that merits more attention in the future.

The very concise empirical question addressed in this note arose in the broader context of including a canonical national IOT within a multiregional framework.

The approach of building a coupled system with global and national multipliers is attractively easy to implement and circumvents a number of challenges inherent in integrating a fresh canonical national IOT into an MRIO. The disadvantage is that second- and higher-order feedback effects do not make use of the canonical national data in such a model. Our results show that for embodied GHG emissions, calculated using the EXIOBASE3 model, this is not a significant disadvantage.

To illustrate with using a case, Edens and colleagues (2015) showed that, for the Netherlands, the difference in total carbon footprint of using superior national data to over-ride the MRIO default yielded a change of 20%, including all effect tiers. We find that just 0.36% of the total Dutch embodied emissions in imports, and 0.1% of total Dutch CO_2_ footprint, is comprised of feedback emissions. Hence, the main consideration for Netherlands is the accurate representation of the total trade flows and, more precisely, the splitting of re-exports and transit trade from imports undergoing significant transformation domestically^1^ (data that will be available in a canonical IOT), not the representation of emissions multipliers applied to the imports and exports. We therefore suggest that a coupled integration method thus represents a viable alternative to the more complex SNAC procedure.

Naturally, there are many aspects to consider in deciding for a particular type of model coupling, and the question of feedback loops is only of them. These other aspects include sectoral and regional aggregation and balancing to reconcile discrepancies (Steen-Olsen et al. 2014; Rodrigues 2014; Lenzen et al. 2009). These aspects relate, however, to the mechanics of integration, rather than the importance of the integration. The amount of feedback effects captured in a model will depend on the details of how the model is linked (with the handling of [dis]aggregation being a key factor). However, the main conclusion of our study is that feedback effects are overall small, and that result should remain unaffected.

## Footnotes

1 As re-exports and transit trade are not modified by Dutch technology, they give no net effect on the overall footprint size.
